# Efficacy of [^177^Lu]Lu-DOTATATE in metastatic neuroendocrine neoplasms of different locations: data from the SEPTRALU study

**DOI:** 10.1007/s00259-023-06166-8

**Published:** 2023-03-06

**Authors:** Mercedes Mitjavila, Paula Jimenez-Fonseca, Pilar Belló, Virginia Pubul, Juan Carlos Percovich, Amparo Garcia-Burillo, Jorge Hernando, Javier Arbizu, Emilia Rodeño, Montserrat Estorch, Belén Llana, Maribel Castellón, Lina García-Cañamaque, Pablo Gajate, Maria Carmen Riesco, Maria Begoña Miguel, David Balaguer-Muñoz, Ana Custodio, Juana María Cano, Alexandra Repetto, Pilar Garcia-Alonso, Maria Angustias Muros, Jose Luis Vercher-Conejero, Alberto Carmona-Bayonas

**Affiliations:** 1 Department of Nuclear Medicine, Hospital Universitario Puerta de Hierro, Majadahonda, Madrid, Spain; 2 Department of Medical Oncology, Hospital Universitario Central de Asturias, ISPA, Oviedo, Spain; 3 Department of Nuclear Medicine, Hospital Universitario La Fe, Valencia, Spain; 4 Department of Nuclear Medicine, Hospital Clínico Universitario de Santiago, Santiago de Compostela, Spain; 5 Department of Endocrinology and Nutrition, Hospital Universitario Gregorio Marañón, Madrid, Spain; 6 Department of Nuclear Medicine, Hospital Universitario Vall d’Hebron, Barcelona, Spain; 7 Department of Medical Oncology, Hospital Universitario Vall d’Hebron, Vall Hebron Institute of Oncology (VHIO), Barcelona, Spain; 8 Department of Nuclear Medicine, Clínica Universidad de Navarra, Pamplona, Spain; 9 Department of Nuclear Medicine, Hospital Universitario de Cruces, Bilbao, Spain; 10 Department of Nuclear Medicine, Hospital de la Santa Creu i San Pau, Barcelona, Spain; 11 Department of Nuclear Medicine, Hospital Universitario Central de Asturias, Oviedo, Spain; 12 Department of Nuclear Medicine, Hospital Universitario Virgen de la Arrixaca, Murcia, Spain; 13 Department of Nuclear Medicine, Hospital HM Sanchinarro, Madrid, Spain; 14 Department of Medical Oncology, Hospital Universitario Ramón y Cajal, Madrid, Spain; 15 Department of Medical Oncology, Hospital Universitario 12 de Octubre, Madrid, Spain; 16 Department of Nuclear Medicine, Hospital Universitario de Burgos, Burgos, Spain; 17 Department of Nuclear Medicine, Hospital Universitario Doctor Peset, Valencia, Spain; 18 Department of Medical Oncology, Hospital Universitario La Paz, CIBERONC CB16/12/00398, Madrid, Spain; 19 Department of Medical Oncology, Hospital General Universitario de Ciudad Real, Ciudad Real, Spain; 20 Department of Nuclear Medicine, Hospital Universitario Son Espases, Mallorca, Spain; 21 Department of Nuclear Medicine, Hospital Universitario de Getafe, Madrid, Spain; 22 Department of Nuclear Medicine, Hospital Universitario Virgen de las Nieves, Granada, Spain; 23 Department of Nuclear Medicine-PET Unit, Hospital Universitario de Bellvitge - IDIBELL, Barcelona, Spain; 24 Department of Medical Oncology, Hospital Universitario Morales Meseguer, University de Murcia, IMIB, Murcia, Spain

**Keywords:** [^177^Lu]Lu-DOTATATE, Lutathera, Lung, Neuroendocrine tumor, PRRT, Radionuclide therapy

## Abstract

**Background:**

Peptide receptor radionuclide therapy (PRRT) is one of the most promising therapeutic strategies in neuroendocrine neoplasms (NENs). Nevertheless, its role in certain tumor sites remains unclear. This study sought to elucidate the efficacy and safety of [^177^Lu]Lu-DOTATATE in NENs with different locations and evaluate the effect of the tumor origin, bearing in mind other prognostic variables. Advanced NENs overexpressing somatostatin receptors (SSTRs) on functional imaging, of any grade or location, treated at 24 centers were enrolled. The protocol consisted of four cycles of ^177^Lu-DOTATATE 7.4 GBq iv every 8 weeks (NCT04949282).

**Results:**

The sample comprised 522 subjects with pancreatic (35%), midgut (28%), bronchopulmonary (11%), pheochromocytoma/ paraganglioma (PPGL) (6%), other gastroenteropancreatic (GEP) (11%), and other non-gastroenteropancreatic (NGEP) (9%) NENs. The best RECIST 1.1 responses were complete response, 0.7%; partial response, 33.2%; stable disease, 52.1%; and tumor progression, 14%, with activity conditioned by the tumor subtype, but with benefit in all strata. Median progression-free survival (PFS) was 31.3 months (95% CI, 25.7–not reached [NR]) in midgut, 30.6 months (14.4-NR) in PPGL, 24.3 months (18.0-NR) in other GEP, 20.5 months (11.8-NR) in other NGEP, 19.8 months (16.8–28.1) in pancreatic, and 17.6 months (14.4–33.1) in bronchopulmonary NENs. [^177^Lu]Lu-DOTATATE exhibited scant severe toxicity.

**Conclusion:**

This study confirms the efficacy and safety of [^177^Lu]Lu-DOTATATE in a wide range of SSTR-expressing NENs, regardless of location, with clinical benefit and superimposable survival outcomes between pNENs and other GEP and NGEP tumor subtypes different from midgut NENs.

**Supplementary Information:**

The online version contains supplementary material available at 10.1007/s00259-023-06166-8.

## Introduction

Somatostatin receptors (SSTRs) are G-protein-coupled receptors with complex biological activities, diffusely distributed in multiple tissues and tumors [1, 2]. Overexpression of SSTRs in more than 80% of well-differentiated gastroenteropancreatic neuroendocrine neoplasms (GEP-NENs) (more in gastrinomas and less in insulinomas), in addition to expression in 50% of bronchopulmonary NENs (BP-NENs), as well as in thyroid tumors, pheochromocytomas and paragangliomas (PPGLs) lay the groundwork for the rationale to study the potential antitumor effect of targeted therapies with radioligands [3, 4].

Under the aegis of this strategy, theragnosis based on SSTRs for both the diagnosis and treatment with peptide receptor radionuclide therapy (PRRT) is one of the most promising treatment approaches in NENs. In vivo expression of SSTRs is detected by SSTR imaging (SRI) with great sensitivity, making it possible to select patients for PRRT with [^90^Y][Y-DOTA-DPhe^1^-Tyr^3^] octreotide (DOTATOC) or [^177^Lu][Lu-DOTA^0^-Tyr^3^] octreotate (DOTATATE) [5, 6], by means of standardized methods, such as Krenning criteria [7].

Nonetheless, NENs’ low incidence and tremendous heterogeneity have been a stumbling block to attaining evidence of PRRT’s efficacy in most tumor subtypes [8, 9]. With a biological imprint modulated by tumor origin, the embryogenesis of the diffuse neuroendocrine system accounts for the broad spectrum of these tumors: GEP-NENs (approximately two thirds of all cases), BP-NENs (22–27%), unknown primaries (10–20%), and up to 5% located in endocrine glands, endocrine islets other than the pancreas (thyroid), and in other organs, such as the gonads [9–12].

The most robust evidence in favor of PPRT in NENs derives from the phase 3 NETTER-1 randomized clinical trial (RCT) comparing [^177^Lu]Lu-DOTATATE and octreotide vs high-dose octreotide in SSTR+, advanced midgut NEN in progression on somatostatin analog (SSA) [13]. The study showed signs of unmistakable efficacy, exhibiting an objective response rate (ORR) of 18% vs. 3% and mature median progression-free survival (mPFS) of 28.4 vs. 8.5 months (HR 0.21; 95% confidence Interval (CI), 0.14–0.33; *p* < 0.0001) with [^177^Lu]Lu-DOTATATE vs. high-dose octreotide, respectively. In the final analysis, median overall survival (mOS) was 48 vs. 36.3 months (HR 0.84; 95% CI, 0.60–1.17; *p* = 0.30) conditioned by the crossover in 36% and probably, by the impact of successive therapies [14]. The evidence is far less resounding for NENs of other locations or those having a worse prognosis. Thus, the FDA/EMA’s approval of [^177^Lu]Lu-DOTATATE in pancreatic NENs (pNENs) was grounded on a non-randomized cohort in which ORRs were documented of 31%, 55%, and 30%; mPFS of 30 (95% CI, 52–68), 30, and 20 months, and mOS of 60 (95% CI, 52–68), 71 (56–86), and 52 months (49–55), in midgut NENs (*n* = 181), pNENs (*n* = 133), and BP-NENs (*n* = 23), respectively [15]. Despite the fact that the regulators considered that a consistent effect across the entire spectrum of SSTR+ well-differentiated GEP-NENs was plausible, leading to unrestricted approval, the truth is that there is still a paucity of evidence for a substantial percentage of factual indications.

More recently, the phase 2 CONTROL NET trial has yielded more solid signals of efficacy in pNENs in a randomized setting with the peculiarity that PRRT was coupled with chemotherapy in the experimental arm in the pNEN cohort [16]. Consequently, the need for more solid information is pressing, inasmuch as pNENs appear to respond well to PRRTs, but later progress somewhat more quickly than midgut NENs [15, 17–20]. It will take other ongoing phase 3 RCTs, such as NETTER-2 (NCT03972488) and COMPETE (NCT03049189), years to yield hard, mature data regarding the role of PPRTs in pNENs. Evidence concerning BP-NEN, PPGLs, subtypes of rarer NENs, or high-grade tumors is even more preliminary [21].

Moreover, the lack of randomized data leaves the issue up in the air of to what point PRRT-based theragnosis should be established in the repertoire of treatments for advanced unresectable NENs independently of their location. This gap is generally more important in daily care, with non-selected patients having a more unfavorable profile compared to clinical trials.

Under these premises, our study (NCT04949282) contributes to the body of evidence available, traditionally sparse in *apropos* of advanced NENs, with this analysis of a national registry of PRRT in which the results of subjects with NENs originating within or outside the digestive tract have been meticulously examined.

## Method

### Study design and population

SEPTRALU (NCT04949282) is a national registry of tumors treated with PRRT sponsored by the Spanish Society of Nuclear Medicine and Molecular Imaging (SEMNIM) in collaboration with the Spanish Society of the Endocrinology and Nutrition (SEEN). The data loggers are nuclear medicine physicians, medical oncologists, endocrinologists, and surgeons from 24 Spanish hospitals.

A registerable case is defined as any adult (> 18 years) with a metastatic, unresectable, SSTR-overexpressing, histologically confirmed neoplasm, that receives at least one cycle of [^177^Lu]Lu-DOTATATE, in accordance with the clinical practice of each center. At all centers, [^177^Lu]Lu-DOTATATE was administered at a dose of 7.4 GBq iv per cycle, in 4 cycles with an interval of 8–10 weeks together with an amino acid solution to protect the kidneys. The sample also included undifferentiated or/and grade 3 (Ki67 > 20%) NENs if they expressed SSTRs. Individuals were excluded if they had < 3 months of follow up except for those who had died during this period. In cases pretreated with PRRTs, outcomes of the first treatment received were evaluated.

The data are managed by means of a website (http://www.septralu.es/) consisting of filters and an online monitoring system to guarantee data reliability and to control missing or inconsistent data (MM and PJF).

The protocol and study were approved by the Spanish Agency of Medicines and Medical Devices (AEMPS) (CSV: DSRZJ6QF1B), a reference Research Ethics Committee, the local agencies and Ethics Committees of each center. The study was conducted in accordance with the Guide of Good Clinical Practices of the International Conference of Harmonization, the principles of the Declaration of Helsinki, and local laws and regulations. All patients still alive at the time of data collection gave their informed consent in writing.

### Endpoints, variables, and assessments

The populations of interest comprised midgut, pancreatic, and other gastroenteropancreatic tumors (collectively, GEP-NENs); as well as BP-NENs, PPGLs, and other non-gastroenteropancreatic tumors (collectively, NGEP-NENs). Midgut tumors included primary jejunum, ileum, appendix, and proximal colon tumors. The group of “other GEP-NENs” collectively included tumors originating in esophagus, stomach, duodenum, biliary, distal colon, rectum, and anus. In addition, neoplasms of unknown primary were clustered as “other GEP-NENs” in the presence of at least one histopathologic marker compatible with digestive origin; if not, as “NGEP-NENs” [22].

The objective was to describe the outcomes (ORR, mPFS, and mOS) and safety (toxicity) of [^177^Lu]Lu-DOTATATE in NGEP-NENs and GEP-NENs, both aggregated and individually by tumor subtype. As a secondary objective, the effect of tumor site was explored, taking into account other prognostic variables and confounding factors.

SRI positivity was graded according to Krenning’s criteria [7], considering positivity when the uptake intensity of the primary tumor and metastases exceeded the uptake of normal liver using any SRI modality. Clinical, treatment, and disease status data were acquired from clinical history, patient interview, and local procedures, which included radiological studies, SRI, and serum and urinary markers that were performed following clinical practice.

The data evaluated included demographic (age, sex), clinical (Eastern Cooperative Oncology Group performance status (ECOG-PS), and symptoms), tumor (primary tumor site, number and location of metastases, functioning, serum, and urinary markers), histopathological (grade, Ki67, differentiation, immunohistochemistry), prior treatments (types, number, time on treatment, and best response), and treatment characteristics (dose, cycles, reasons for discontinuation, ORR, mPFS, mOS, toxicity) information. Response was assessed on the basis of morphological criteria, ORR (proportion of subjects with partial or complete response according to RECIST1.1 criteria on computed tomography (CT) or magnetic resonance (MR)), and functional criteria based on SRI ([^68^Ga]Ga-DOTA-Tyr-octreotide PET/CT, [^111^In]In-DTPA0-D-Phe1-octreotide SSTR scintigraphy or post-therapy [^177^Lu]Lu-DOTATATE) scans. All patients were re-evaluated by post-therapy scans at the end of treatment; assessment by CT or PET-CT was performed according to each center’s clinical practice (generally every 3–6 months). The best response achieved at follow-up was determined and the measurement technique was the one used locally, as per clinical practice in each center. In the case of PET-CT scans, response was graded analogously to the PERCIST criteria, with partial response defined as a reduction in lesion size or intensity of ≥ 30% (minimum absolute change in peak standardized uptake value [SUV] of 0.8), complete response defined as normalization of all SUVs, and progression defined as an increase in intensity > 30% or the appearance of new lesions, verified through morphological criteria during follow-up [23]. In the case of scintigraphy evaluation, a semi-quantitative assessment of response was requested through the relationship between tumor uptake intensity and reference zones (liver or spleen), with response or progression defined as an increase or decrease in uptake of approximately 30%. Biochemical response was evaluated utilizing the criteria proposed by the Italian Trials in Medical Oncology (ITMO) [24]. Partial response was defined as a ≥ 50% decrease in plasma Chromogranin A (CgA), 5-hydroxyindoleacetic acid, or other secreted biomarkers compared to the baseline, stable disease was defined as a decrease of < 50% or an increase of < 25%, and progressive disease (PD) was characterized by an increase of ≥ 25%. Moreover, the greatest reduction in biomarkers from baseline to radiological progression was also documented. The response was categorized based on the stability of the biomarker concentration during the follow-up period. In the case of symptomatic response, data were obtained from the clinical history, and subsequent follow-up. Investigators were asked to report documentary evidence of subjective improvement in various areas such as functional syndrome due to hormonal hypersecretion (e.g., flushing or diarrhea), constitutional syndrome, functional improvement, or improvement in specific symptoms such as pain or gastrointestinal clinical symptoms. mPFS and mOS were defined as the period between the date that [^177^Lu]Lu-DOTATATE was initiated and progression according to the investigator’s assessment (mPFS) or death due to any cause (mOS), censoring those cases without an event at the time of the last follow-up. Assessments were performed during each treatment cycle and thereafter, following standard practice, at least every 6 months until progression or demise. Adverse events were classified according to the National Cancer Institute Common Toxicity Criteria (NCI-CTC), version 4.03, considering the maximum toxicity developed during follow up.

### Statistical analyses

The time-to-event variables (mPFS/mOS) were evaluated utilizing the Kaplan-Meier estimator, comparing survival functions via log-rank tests. The results were modeled using multivariable Cox proportional hazards regression. The covariables selected in these models were chosen by theoretical criteria, consistent with the review of the literature and registry coordinators (MM, PJF, ACB, JCP), while avoiding collinearity (variation inflation factor < 2.5). For this purpose, the most common, known prognostic factors in NENs that might act as confounding factors were taken into account. The model was designed to assess the effect of tumor subtype while controlling for various confounding variables, so coefficients associated with confounders should not be interpreted as implying causality [25].

Missing values were handled by multiple imputation with predictive mean matching by chained equations, discarding covariates with > 20% of missing data [26]. The study had a fixed sample size, contingent on the number of registered cases, which means that the inferences had to be interpreted as a function of the magnitude of the CIs. However, to specify the multivariable models, the “rule of thumb” of having at least 15 events per degree of freedom spent (between 15 and 16 in this context) was applied [27]. The correlation between PFS and OS was quantified using Kendall’s τ associated with Hougaard’s copula models for bivariate survival data [28]. Descriptive data were treated with appropriate standard statistics and measures in each instance. Proportions were compared by χ2-tests. The outcomes for uncommon neoplasms were reported individually using swimmer plots.

## Results

### Baseline characteristics

The registry contains 562 eligible patients treated between June 2014 and June 2022, 522 of whom were eligible given that follow-up data were available for them. The sample consisted of 35% pNENs (*n* = 182), 28% midgut NENs (*n* = 148), 11% BP-NENs (*n* = 56), 6% PPGLs (*n* = 31), 11% other GEP-NENs (*n* = 60), and 9% other NGEP-NENs (*n* = 45). Baseline characteristics by tumor subtype are reported in Table 1 and aggregated into GEP-NENs and NGEP-NENs are provided in Supplementary Materials, Annex Table 1. Median age was 60 years (range, 21–88) and 60.2% (*n* = 314) were male. Most neoplasms were well-differentiated (90%, *n* = 470), with median Ki67 of 5% (range, 0–80), and Krenning score of 3 (uptake exceeding hepatic) in 75.7% (*n* = 395). Tracer uptake varied with histologic grade but was independent of line of treatment (Supplementary Materials, Annex Table 2). The percentage of Krenning 4 tumors was higher in patients diagnosed by [^68^Ga]Ga-DOTATOC vs. SSTR scintigraphy (33% vs. 14%, *p* < 0.0001) (Supplementary Materials, Annex Table 2). Roughly one-third were functioning tumors. Treatments were given as first, second, third, and subsequent lines in 4.2% (*n* = 22), 35.2% (*n* = 184), 29.6% (*n* = 155), and 30.8% (*n* = 161), respectively. Most frequent previous therapies were somatostatin analog (91%), everolimus (42.9%, *n* = 224), or chemotherapy (27.6%, *n* = 144) with no differences between GEP and NGEP-NENs. The most substantial differences by tumor subtypes were younger age, greater prevalence among females, and more bone metastases in PPGLs; the predominance of liver, peritoneal, and lymph node involvement in midgut tumors, and the preponderance of males in BP-NENs (Table 1).

**Table 1:** Baseline characteristics by tumor subtype

Baseline characteristic	All patients, *N* = 522 (100%)	pNEN, *N* = 182 (100%)	Midgut NEN, *N* = 148 (100%)	BP-NEN, *N* = 56 (100%)	PPGLs, *N* = 31 (100%)	Other GEP-NEN, *N* = 60 (100%)	Other NGEP-NEN, *N* = 45 (100%)
Gender							
Men	314 (60.2)	105 (57.7)	93 (62.8)	44 (78.6)	14 (45.2)	33 (55.0)	25 (55.6)
Women	208 (39.8)	77 (42.3)	55 (37.2)	12 (21.4)	17 (54.8)	27 (45.0)	20 (44.4)
Age (years), median (range)	60 (21–88)	59 (22–84)	65 (38–88)	60 (21–83)	50 (21–78)	61 (21–85)	60 (29–81)
ECOG performance status							
0	256 (49.0)	103 (56.6)	71 (48.0)	24 (42.9)	10 (32.3)	28 (46.7)	20 (44.4)
1	210 (40.2)	63 (34.6)	59 (39.9)	26 (46.4)	15 (48.4)	26 (43.3)	21(46.7)
2	37 (7.1)	13 (7.1)	11 (7.4)	5 (8.9)	4 (12.9)	3 3 (5.0)	1 (2.2)
> 2	4 (0.8)	0	1 (0.7)	0	1 (3.2)	11 (1.7)	1 (2.2)
Unknown	15 (2.9)	3 (1.6)	6 (4.1)	1 (1.8)	1(3.2)	22 (3.3)	2 (4.4)
Ki-67%, median (range)	5 (0–80)	6 (1–60)	3 (1–35)	6 (1–30)	4 (0–20)	5 (1–80)	10 (1–77)
Missing	95 (18.2)	32 (17.6)	18 (12.2)	12 (21.4)	18 (58.1)	6 (10.0)	9 (20.0)
WHO 2017							
NET G1	178 (34.1)	51 (28.0)	64 (43.2)	15 (26.8)	13 (41.9)	23 (38.3)	12 (26.7)
NET G2	292 (55.9)	103 (56.6)	79 (53.4)	35 (62.5)	16 (51.6)	34 (56.7)	25 (55.6)
NET G3	42 (8.0)	24 (13.2)	4 (2.7)	3 (5.4)	2 (6.5)	2 (3.3)	7 (15.6)
NEC G3	10 (1.9)	4 (2.2)	1 (0.7)	3 (5.4)	0	1 (1.7)	1 (2.2)
Hormonal syndrome	169 (32.4)	36 (19.8)	73 (49.3)	10 (17.9)	20 (64.5)	16 (26.7)	14 (31.1)
Localization of metastases							
Liver	436 (83.5)	168 (92.3)	132 (89.2)	47 (83.9)	9 (29.0)	50 (83.3)	30 (66.7)
Lymph nodes	286 (54.8)	94 (51.6)	91 (61.5)	30 (53.6)	22 (71.0)	23 (38.3)	26 (57.8)
Peritoneum	90 (17.2)	17 (9.3)	52 (35.1)	1 (1.8)	2 (6.5)	8 (13.3)	10 (22.2)
Bone	145 (27.8)	35 (19.2)	25 (16.9)	30 (53.6)	20 (64.5)	17 (28.3)	18 (40.0)
Lung	48 (9.2)	7 (3.8)	4 (2.7)	14 (25.0)	8 (25.8)	9 (15.0)	6 (13.3)
Other	78 (14.9)	22 (12.1)	17 (11.5)	15 (26.8)	1 (3.2)	13 (21.7)	10 (22.2)
Prior surgery							
Primary tumor	281 (53.8)	83 (45.6)	94 (63.5)	34 (60.7)	26 (83.9)	31 (51.7)	13 (28.9)
Metastases	121 (23.2)	49 (26.9)	41 (27.7)	7 (12.5)	4 (12.9)	14 (23.3)	6 (13.3)
Number of prior systemic treatments							
0	22 (4.2)	4 (2.2)	1 (0.7)	2 (3.6)	12 (38.7)	0	3 (6.7)
1	184 (35.2)	55 (30.2)	73 (49.3) 48	12 (21.4)	6 (19.4)	22 (36.7)	16 (35.6)
2	144 (29.7)	55 (30.2)	(32.4)	21 (37.5)	5 (16.1)	16 (26.7)	10 (2.2)
> 2	161 (30.8)	68 (37.4)	26(17.6)	21 (37.5)	8 (5.8)	22 (36.7)	16 (35.6)
Prior systemic treatments							
Somatostatin analogues	477 (91.4)	168 (92.3)	146 (98.6)	53 (94.6)	14 (45.2)	57 (95.0)	39 (86.7)
Chemotherapy	144 (27.6)	70 (38.5)	15 (10.1)	16 (28.6)	14 (45.2)	11 (18.3)	18 (40.0)
Everolimus	224 (42.9)	78 (42.9)	59 (39.9)	32 (57.1)	3 (9.7)	20 (44.4)	20 (44.4)
Sunitinib	104 (19.9)	61 (33.5)	10 (6.8)	7 (12.5)	4 (12.9)	12 (20)	10 (22.2)
Other tyrosin quinase inhibitor	54 (10.3)	15 (8.2)	15 (10.1)	10 (17.9)	3 (9.7)	6 (10.0)	5 (11.1)
Prior locoregional and ablative therapies	77 (14.8)	36 (19.8)	18 (12.2)	4 (7.1)	0	15 (25.0)	4 (8.9)
Median time from initial diagnosis to PRRT,months (range)	40.6 (0–288)	40.7 (0–276)	39.0 (2–288)	39.7 (3–273)	41.2 (1–169)	41.4 (2–186)	40.9 (2–127)
Median time from most recent progression until PRRT, months (range)	2.3 (0–92.1)	2.2 (0.1–33)	2.7 (0–92)	2.4 (0–16.9)	2.9 (0.9–16)	2.2 (0.5–31)	2.2 (0.6–25)
SRI, Krenning scale							
2	44 (8.4)	15 (8.2)	12 (8.1)	4 (7.1)	2 (6.5)	1 (1.7)	10 (22.2)
3	395 (75.7)	133 (73.1)	120 (81.1)	41 (73.2)	21 (67.7)	51 (85.0)	29 (64.4)
4	83 (15.9)	34 (18.7)	16 (10.8)	11 (19.6)	8 (25.8)	8 (13.3)	6 (13.3)
[^18^F]F-FDG PET-CT							
Not done	383 (73.4)	130 (71.4)	122 (82.4)	41 (73.2)	13 (41.9)	50 (83.3)	27 (60.0)
Consistent with SRI	50 (9.6)	16 (8.8)	6 (4.1)	7 (12.5)	12 (38.7)	2 (3.3)	7 (15.6)
Not concordant	89 (17.0)	36 (19.8)	20 (13.5)	8 (14.3)	6 (19.4)	8 (13.3)	11 (24.4)

*pNEN* pancreatic neuroendocrine neoplasm, *NEN* neuroendocrine neoplasm, *BP*-*NEN* bronchopulmonary neuroendocrine neoplasm, *GEP*-*NEN* gastroenteropancreatic neoplasm, *NGEP*-*NEN* no gastroenteropancreatic neoplasm, *PPGL* pheochromocytoma and paraganglioma, *ECOG* Eastern Cooperative Oncology Group, *WHO* World Health Organization, *PRRT* peptide receptor radionuclide therapy, *PET* positron emission tomography, *SRI* somatostatin receptor-based imaging, *18F*-fluorodeoxyglucose position emission tomography-computed tomography

**Table 2:** Response assessment according to tumor subtype in subjects with measurable and response-assessable disease

Tumor subtype	PD, *N* (%)	DC, *N* (%)	SD, *N* (%)	PR, *N* (%)	CR, *N* (%)	Total, *N* (%)
pNENs	24 (15.2)	134 (84.8)	67 (42.4)	66 (41.8)	1 (0.6)	158 (100)
Midgut NENs	8 (6.5)	116 (93.5)	81 (65.3)	34 (27.4)	1 (0.8)	124 (100)
BP-NENs	11 (22.4)	38 (77.6)	24 (49.0)	14 (28.6)	0	49 (100)
PPGLs	4 (15.4)	22 (84.6)	17 (65.4)	5 (19.2)	0	26 (100)
Other GEP-NENs	7 (14.6)	41 (85.4)	24 (50.0)	17 (35.4)	0	48 (100)
Other NGEP-NENs	8 (21.1)	30 (78.9)	18 (47.4)	11 (28.9)	1 (2.6)	38 (100)
Total	62 (14.0)	381 (86.0)	231 (52.1)	147 (33.2)	3 (0.7)	443 (100)

*pNEN* pancreatic neuroendocrine neoplasm, *BP*-*NEN* bronchopulmonary neuroendocrine neoplasm, *PPGL* pheochromocytoma and paraganglioma, *NEN* neuroendocrine neoplasia, *GEP* gastroenteropancreatic, *NGEP* no gastroenteropancreatic, *PD* progression disease, *DC* disease control (CR+PR+SD), *SD* stable disease, *PR* partial response, *CR* complete response

Note: Response was evaluated using RECIST v1.1 criteria

### Treatment

At the time of analysis, 90% (*n* = 471) had completed therapy with [^177^Lu]Lu-DOTATATE. The most common reasons for withdrawal were having completed the schedule planned in 74% (*n* = 385), progression in 7% (*n* = 37), or toxicity in 3% (*n* = 15) of the subjects. Twenty-one intra-treatment deaths (4%) were recorded in relation to tumor-associated complications, 71% of which occurred following progression to ≥ 2 previous lines. Of the participants who completed therapy, 94% received the four standard doses; the rest, 5–8 doses, with no variation based on tumor subtype (the reason for administering > 4 doses was usually retreatment after 18–60 months of initial therapy, except in 2 cases that initially received 6 cycles to increase tumor regression). Almost all (97%) of the doses were 7.4 GBq and average interval between them was 2.1 months (90% between 1.7 and 3 months). Median time from diagnosis of metastasis until PRRT was 40.6 months (range, 0–288). Prior to [^177^Lu]Lu-DOTATATE, 94% (*n* = 489) displayed tumor progression as per RECIST.

### Response, survival, and toxicity outcomes

**Fig. 1:**
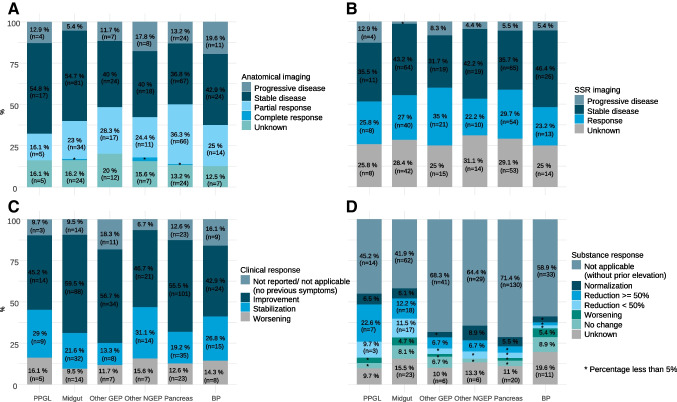
Response rate, assessed with anatomical imaging (**A**), SSR imaging (**B**), clinical interview (**C**), and with markers (**D**). Bivariate χ2 tests of response and tumor type, with SSR imaging: χ2 = 15.33, degrees of freedom [d.f.] = 15, *p* value = 0.4277; anatomical: χ2 = 29.33, d.f. = 20, *p* value = 0.0816; clinical: χ2 = 15.64, d.f. = 15, *p* value = 0.4047; and biomarkers: χ2 = 13.86, d.f. = 15, *p* value = 0.5359

Response based on radiological, SRI, clinical, or biochemical criteria was available in 85%, 72%, 90%, and 87% of the cases, respectively. Response distribution can be seen in Fig. 1. Considering only subjects with measurable and response-evaluable disease (443/552), the best RECIST 1.1 response was complete response in three (0.7%), partial response in 147 (33.2%), stable disease in 231 (52.1%), and tumor progression in 62 (14%). The overall disease control rate (responses and stabilizations) was 86% (*n* = 381). ORR and disease control rate broken down by tumor subtype was 42.4% and 84.8% in pNENs, 35.4% and 85.4% in other GEP-NENs, 31.5% and 78.9% in other NGEP-NENs, 28.6% and 77.6% in BP-NENs, 28.2% and 93.5% in midgut, and 19.2% and 84.6% in PPGLs (χ2 = 27.1, degrees of freedom [d.f.] = 15, *p* = 0.0274) (Table 2). The landmark-analysis survival curves stratified by response are shown in Supplementary Materials, Annex Fig. 1 . The response type prior to 12-month landmark predicts OS with a concordance index of 0.646 (standard error [SE] = 0.034). Clinical responses were higher than radiographic or SRI responses across all tumor types (Fig. 1). The response rate tended to decrease with the number of previous lines, with the exception of midgut NENs (Supplementary Materials, Annex Table 3). No substantial differences were detected across tumor subtypes for SRI, clinical, or biochemical response (Fig. 1, see χ2 tests in the footnote). The response stratified by tumor grade is shown in Table 4, with more partial responses but also a higher rate of tumor progression, and lower percentage of stable disease in high-grade tumors (G3) versus NET G1/2 (Supplementary Materials, Annex Table 4).

**Table 3:** Multivariable Cox PH regression for progression-free survival

Variable	HR	(95% CI)	*p* value
Number of previous lines	1.22	1.00- 1.50	0.048
Everolimus	1.38	1.01-1.89	0.040
Ki67%	1.22	1.08-1.39	0.001
ECOG performance status			
0	Ref.	Ref.	—
1	1.81	1.36–2.41	< 0.0001
2	2.58	1.64–4.05	< 0.0001
Tumor site			
pNEN	Ref.	Ref.	–
Midgut NEN	0.64	0.44–0.93	0.020
BP-NEN	0.89	0.73–1.73	0.585
PPGL	1.12	0.49–1.62	0.713
Other GEP-NEN	0.69	0.44–1.09	0.118
Other NGEP-NEN	0.68	0.41–1.13	0.144
Krenning score			
2	Ref.	Ref.	Ref.
3	0.73	0.46–1.15	0.183
4	0.53	0.30–0.92	0.025
Metastases			
Liver	Ref.	Ref.	Ref.
Extrahepatic	1.39	1.04–1.87	0.025
Extranodal	1.14	0.48–2.71	0.751

*PFS* progression-free survival, *HR* hazard ratio, *CI* confidence interval, *Ref*. reference, *ECOG* Eastern Cooperative Oncology Group, *pNEN* pancreatic neuroendocrine neoplasm, *BP*-*NEN* bronchopulmonary neuroendocrine neoplasm, *PPGL* pheochromocytoma and paraganglioma, *NEN* neuroendocrine neoplasm, *GEP* gastroenteropancreatic, *NGEP* no gastroenteropancreatic

**Table 4:** Adverse events

Adverse events	All patients *N* = 522 (100%)	
Drug-related adverse events	All grades	Grade 3/4
Nausea	30.4%	0.6%
Vomiting	19.5%	0.2%
Hematological toxicity	29.8%	4.7%
Asthenia	13.0%	0.7%
Alopecia	7.2%	
Enteritis	4.4%	0.1%
Nephrotoxicity	3.6%	0.9%
Abdominal pain	3.0%	0.1%
Urticaria	2.3%	–
Hepatotoxicity	1.3%	0.7%
Decreased appetite	1.3%	–
Flushing	0.9%	–
Fever	0.7%	0.1%
Mucositis	0.3%	–
Musculoskeletal pain	0.3%	–
Skin disorders	0.1%	–
Others	2.1%	0.3%
Death		0.9%

**Fig. 2:**
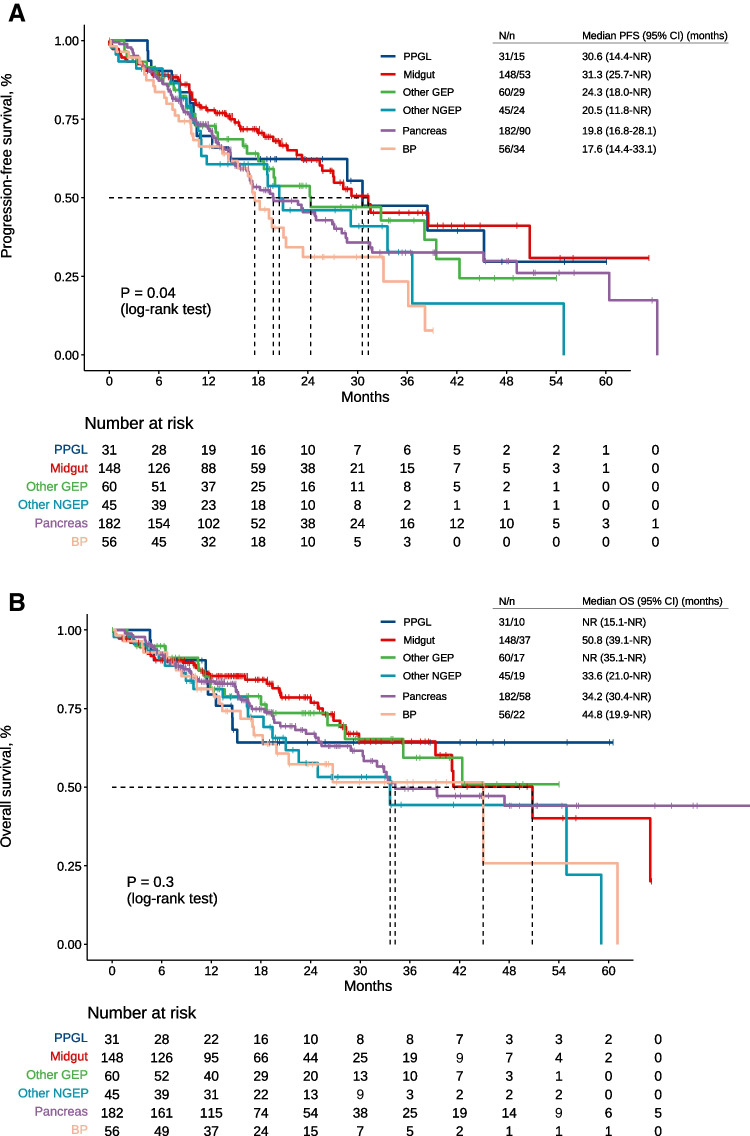
Kaplan-Meier curves for progression-free survival (**A**) and overall survival (**B**) based on tumor site. *N*/*n* sample size/events, *PFS* progression-free survival, *CI* confidence interval, *NR* not reached, *PPGL* pheochromocytoma and paraganglioma, *GEP* gastroenteropancreatic, *NGEP* non-gastroenteropancreatic, *BP* bronchopulmonary

After a median follow up of 21.2 months in participants still alive, 245 progressions to PRRT events and 163 death events were recorded, with mPFS of 24.3 (95% CI, 20.6–28.7) and mOS of 42.3 (95% CI, 34.2–61.1) months. Survival results stratified by tumor subtype are reflected in Fig. 2. mPFS was 31.3 months (95% CI, 25.7–not reached [NR]) in midgut, 30.6 months (14.4-NR) in PPGLs, 24.3 months (18.0-NR) in other GEP-NENs, 20.5 months (11.8-NR) in other NGEP-NENs, 19.8 months (16.8–28.1) in pNENs, and 17.6 months (14.4–33.1) in BP-NENs (Fig. 2A). In any of the strata, results worsened with increasing number of previous treatments (Supplementary Materials, Annex Table 5). However, the correlation between PFS and OS does not seem to vary significantly based on the number of previous treatments: Kendall’s τ = 0.765 (SE = 0.036), 0.794 (SE = 0.026), and 0.750 (SE = 0.026) for patients who received 0–1, 2, or 3 or more prior therapies for PRRT, respectively.

Sensitivity analysis for PFS by histological grade is found in Supplementary Materials, Annex Table 6. Likewise, it cannot be ruled out that the detection method, [^68^Ga]Ga-DOTATOC vs. SSTR scintigraphy, constitutes an additional source of heterogeneity (Supplementary Materials, Annex Table 6). In the 52 subjects with grade 3 NENs, mPFS increased with SSTR expression in SRI, but the signal was weak (i.e., median PFS of 8.8 vs. 26.9 months in NENs with Krenning 2 vs. 4 [χ2 = 3.6, d.f. = 2, *p* = 0.2]) (Supplementary Materials, Annex Table 2).

Multivariable Cox regression model for PFS is displayed in Table 3. Taking the most numerous stratum (pNENs) as a reference, midgut NENs had less risk of progression (HR for PFS of 0.69; 95% CI, 0.44–0.93; *p* = 0.02).

**Fig. 3:**
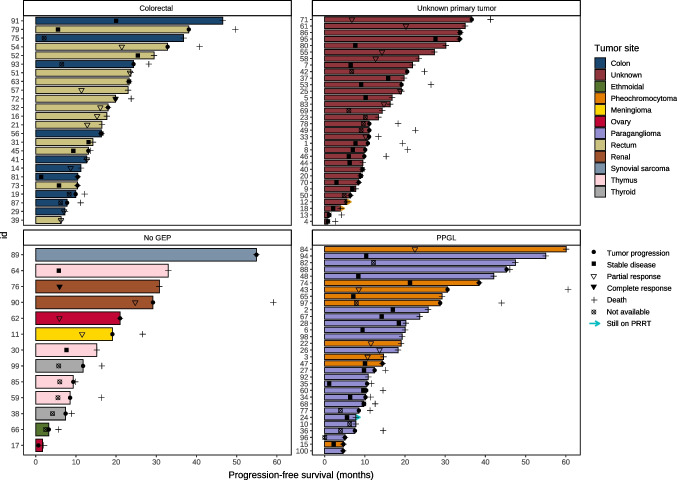
Swimmer plot with results of responses and survival in individual tumors. Each bar represents one patient. *PRRT* peptide receptor radionuclide therapy, *PPGL* pheochromocytoma/paraganglioma, *GEP* gastroenteropancreatic

mOS was 50.8 months (95% CI, 39.1-NR) in midgut NENs, 44.8 months (19.9-NR) in BP-NENs, 34.2 months (30.4-NR) in pNENs, 33.6 months (21.0-NR) in other NGEPs, and not-reached in other GEP-NENs and PPGLs (log-rank test, *p* = 0.3) (Fig. 2B). Individual survival results in rare neoplasms are displayed in a swimmer plot (Fig. 3). Of note is the 57% (8/14) ORR in rectal NENs; tumor control rate (objective response + stable disease) of 72% (23/32) and ORR of 21% in tumors of unknown primary, and tumor control rate of 64% (14/22) and 89% (8/9), in paragangliomas and pheochromocytomas, respectively.

As for safety, [^177^Lu]Lu-DOTATATE was associated with scant severe toxicity, with hematological toxicity the only grade 3–4 side effect presenting an incidence > 1% (4.7%) (Table 4). The most common adverse effects were nausea (30.4%), hematological (29.8%), emesis (19.5%), asthenia (13%), and alopecia (7.2%). In a sensitivity analysis, prior treatment with chemotherapy or biological agent was not associated with increased hematological toxicity (Supplementary Materials, Annex Table 7). Grade 5 events included pancytopenia (in 2 patients), leukemia (in 1 patient), and myelodysplastic syndrome (in 2 patients). In addition, two patients who died due to tumor progression had severe cytopenia attributed to PRRT.

## Discussion

This analysis of the SEPTRALU registry confirms, albeit with nuances, the effect of [^177^Lu]Lu-DOTATATE in metastatic, SSTR-expressing NENs, regardless of location. The results in midgut NENs were similar to those of the NETTER-1 trial [13], and outcomes in BP-NENs, PPGLs, and other neoplasms comparable to that seen in pNENs. The rationale for this study is that therapy and optimal treatment sequence in advanced NENs continues to be poorly established and there is still limited evidence in favor of PRRT in various tumor subtypes. The approval of [^177^Lu]Lu-DOTATATE for SSTR+ GEP-NENs was based on the comparison of the results of the NETTER-1 RCT in ileal tumors and the data from the ERASMUS MC series (Rotterdam) in pNENs. In contrast, with less activity in the Rotterdam series, BP-NENs and other subtypes were excluded from the approval. Uncertainty underlies this criterion due to the smaller size of these strata, as well as the reasonable doubts regarding the external validity of the data derived from a single-center, non-randomized study. This uncertainty has been reflected in disparate clinical recommendations. Thus, the 2016 NEN ENETS clinical guidelines does not reflect PRRT as an alternative for BP-NEN [29], whereas the Commonwealth Neuroendocrine (CommNETs) and North American (NANETS) 2020 clinical guidelines suggest that PRRT can be an option in patients with SSTR+ BP-NEN, on the basis of an expert opinion [30].

Compiling the clinical practice from many centers, the SEPTRALU study has revealed that the ORR of [^177^Lu]Lu-DOTATATE is higher in pNEN vs midgut NEN, which, in turn, is similar for the remaining neoplasms. The results for pNENs in the SEPTRALU registry are slightly less favorable than those reported in other series (ORR 42% vs. 52–60%, mPFS 19.8 vs. 31–34 months, and mOS 34.2 vs. 53–71 months), respectively [15, 18, 19]). The cause for these discrepancies lies in the heterogeneous composition of the samples, with individuals having a worse prognosis and heavily pretreated in the SEPTRALU registry (i.e., 61% and 6% had received somatostatin analogs or chemotherapy prior to PRRT in the Rotterdam series vs. 92% and 38% in our registry, with multiple participants treated with TKIs or everolimus). The greater ORR seen for [^177^Lu]Lu-DOTATATE in pNENs was not clearly correlated with other endpoints. On the other hand, the findings are consistent with those of the recent OCLURANDOM trial, which reported a median PFS of 20.7 months (95% CI 17.2–23.7), similar to the results from our study [31].

In our series, midgut NENs had better mPFS, with an adjusted HR of 0.64 (95% CI, 0.44–0.93) using pNENs as a reference. This datum coincide with other bibliographic reports [20, 32]. For example, a German, multicenter registry with 450 cases reported greater mOS in tumors of the ileum-jejunum vs. other locations (HR 0.39; 95% CI, 0.18–0.87; *p* = 0.021) [32]. This worse survival rate raises questions surrounding the benefit dimension of [^177^Lu]Lu-DOTATATE in the stratum of pNENs and its optimal application, which must be resolved by the ongoing phase 3 RCTs (NCT03972488, NCT03049189).

As in other series, the evidence about other rarer subtypes of NENs is limited, albeit our data point toward an effect that is consistent with the literature. As for BP-NENs, the 28.6% ORR and the mPFS and mOS of 17.6 and 44.8 months, respectively, are in keeping with the data from a small Italian phase 2 trial that reported an ORR of 15%, 18.5-month mPFS (95% CI, 12.9–26.4), and 48.6-month mOS (95% CI, 26.4–68.9) [33]. The remainder of the observational studies have yielded similar results, with ORR of 10–40%, mPFS of 17–28 months, and mOS of 40–59 months [34–38]. However, it is noteworthy that all of these previous series, including the phase 2 trial, have a limited number of patients (< 50), except for the Milan series (*n* = 114) which recruited patients between 1997 and 2012 [34]. Since RCTs and indications for therapeutic alternatives have traditionally focused on GEP-NENs, it becomes increasingly important to have evidence of [^177^Lu]Lu-DOTATATE efficacy in this underserved location. Likewise, in the case of PPGLs, our results (19% ORR and 30.6 months mPFS) are comparable to those of a small Polish phase 2 trial that reported an ORR of 8%, mPFS of 35 months (95% CI, 24.4–93.1), and mOS of 68 months (95% CI, 38.6–105.1) [39]. Similarly, an Italian study reported a mPFS and mOS of 27.5 months (95% CI, 14.5–51.5) and 142.6 months (95% CI 76.1–146.2), respectively in sympathetic paragangliomas [40]. Moreover, a meta-analysis of 12 studies on PRRT in SSTR+ advanced PPGL reported a pooled estimate of 25% ORR (95% CI, 19–32%), 61% clinical response (30–88%), and 64% biochemical response (11–96%). The mPFS was 37.1 months (95% CI, 32.1–42.0 and range, 10–91) and mOS was 54.5 months (42.5–66.5) [41]. In this context, it is important to note the remarkable disease control rate observed in PPGLs (84.6%) with a median PFS that is comparable to that reported in midgut tumors. These results may have implications for the management of these conditions; however, further validation through larger-scale prospective trials is required to fully establish its significance.

Our study also endorses the efficacy of [^177^Lu]Lu-DOTATATE in uncommon NENs, for which the evidence is even scarcer, such as hindgut NENs and other NGEP-NENs, including those of unknown origin that cannot be mapped in the digestive tract. Activity has been detected in all of them and deserves to be confirmed in future RCTs. The similar prognosis of NENs of unknown primary or other locations compared to pNENs has also been reported in a series of 1048 patients treated at a German reference center [20]. Therefore, our study indicates that the benefit of PRRT in SSTR+ tumors is independent of tumor site in the extra-midgut scenario, casting doubts on decision making based on this criterion.

In this series, hematological toxicity and alopecia are in keeping with published findings of the NETTER-1 RCT, but nausea/vomiting and asthenia are much lower (less than half), possibly because the centers are aware of these side effects and therefore prevent them.

As for generalizability, it must be remembered that the SEPTRALU registry cases had a metastatic, SSTR+ (defined by a Krenning score of 2-4) NEN, in progression (94%), and of any location. Unlike the NETTER-1 RCT [13], this registry includes 71% of non-midgut NENs; 10% with Ki67 > 20%; 60.5% treated in third or successive lines; patients and NENs having a worse prognosis, which must be contemplated when making treatment decisions.

Our work has several limitations, not least of which are its retrospective nature and the relative immaturity of the follow up (i.e., events of progression recorded in 245/522). SRIs were conducted according to the imaging modalities and laboratory tests available at each center. Readers should be aware that both functional response evaluation by scintigraphy and biochemistry were performed in a semi-quantitative or non-standardized manner, with the potential loss of sensitivity that this implies. Similarly, symptomatic response was evaluated through records in medical histories and subsequent follow-up, without a validated questionnaire, which limits the accuracy of the data and prevents precise investigation into specific aspects of quality of life. The readers should also be aware that the multivariable model in Table 3 attempts to assess the effect of tumor subtype, while considering various confounding factors. The choice of everolimus as a covariate is based solely on its widespread utilization among NENs, and the results should not be interpreted as implying causal effects [25]. PFS estimation may also have been affected by the local regularity in performing CTs. Furthermore, other uncommon factors may be relevant in unusual contexts (i.e., the effect of SDHB mutation or PPGL subtype) [39, 40, 42].

With these limitations present, our article contains noteworthy information that is applicable in clinical practice, and it contribute to externally validate the results of other studies such as the OCLURANDOM trial [31]. To begin with, PRRT continues to be the modality associated with greater ORRs in well-differentiated midgut NENs and potentially has an ORR similar to chemotherapy in other scenarios of extramidgut NENs. Nevertheless, our data point toward decreased efficacy as the number of previous lines increases. Treatment sequence clarification by RCTs notwithstanding, the finding might be related with non-differentiation and the tumors being less dependent on SSTR, which would indicate the advisability of prescribing PRRT earlier [20, 43–45]. PRRT is currently indicated following progression to somatostatin analog, pending the results of the COMPETE RCT, comparing everolimus to PRRT, and of the NETTER2 study, comparing somatostatin analog vs. PRRT in first line, to have more evidence with respect to the best sequence [14, 46]. However, it is noteworthy that PFS appears to be a reliable surrogate for OS, regardless of the line in which PRRT is administered. This suggests that PRRT effectively controls the disease and contributes significantly to the patient’s survival, while the impact of subsequent treatments is likely to be less significant. Second, in the case of PPGLs, treatment choice should be informed by the concentration of the tracer in the various molecular imaging modalities, being mindful of the greater experience with [^131^I]I-metaiodobenzylguanidine. However, PRRT has the potential advantage of producing fewer hematological side effects and not requiring thyroid blockade prior to treatment. Third, our data demonstrate that PPRT is active in grade 3 NENs with an ORR comparable to that of well-differentiated tumors, albeit mPFS is more limited. Fourth, an evaluation of both [^18^F]fluoro-2-deoxy-d-glucose PET-CT and SRI might help to select patients with grade 3 NENs who can benefit more with PRRT, despite the evidence being weak. Subject to the presence of SSTRs demonstrated by SRI, PRRT prescription may be particularly suitable in symptomatic individuals that need rapid relief.

As for safety, our data point toward [^177^Lu]Lu-DOTATATE having a favorable profile, with scant adverse events that were generally mild. Thus, the literature reflects that [^177^Lu]Lu-DOTATATE is safer than [^90^Y]Y-DOTATOC thanks to the lower doses absorbed by the kidneys and bone marrow at comparable dosages and to the longer half-life (6.7 versus 2.7 days) [36].

In short, our data imply that [^177^Lu]Lu-DOTATATE is active and safe in wide range of NENs, with both radiological and clinical response rates and survival outcomes in pNENs and other tumor subtypes coinciding, which would support its tumor-agnostic use in the presence of SSTRs demonstrated by SRI techniques.

## Supplementary Information

Below is the link to the electronic supplementary material.

Supplementary file1 (DOCX 18 KB)

Supplementary file2 (DOCX 16 KB)

Supplementary file3 (DOCX 18 KB)

Supplementary file4 (DOCX 13 KB)

Supplementary file5 (DOCX 15 KB)

Supplementary file6 (DOCX 17 KB)

Supplementary file7 (DOCX 14 KB)

Supplementary file8 (PDF 25 KB)

## Data Availability

All the data generated or analyzed in this study are included in the manuscript, tables, and figures.

## Abbreviations

[^177^Lu]Lu-DOTATATE: [^177^Lu][Lu-DOTA^0^, Tyr^3^] Octreotate (DOTATATE)
[^90^Y]Y-DOTATOC: [^90^Y][Y-DOTA-DPhe^1^-Tyr^3^] octreotide
[^68^Ga]Ga-DOTATOC: [^68^Ga]Ga-DOTA-Tyr-octreotide
AEMPS: Spanish Agency of Medicines and Medical Devices
BP-NENs: Bronchopulmonary neuroendocrine neoplasms
CI: Confidence interval
CT: Computed tomography
DF: Degrees of freedom
ECOG-PS: Eastern Cooperative Oncology Group performance status
EMA: European Medicines Agency
FDA: Food and Drug Administration
GBq: Gigabecquerel
GEP: Gastroenteropancreatic
GEP-NENs: Gastroenteropancreatic neuroendocrine neoplasms
HR: Hazard ratio
IV: Intravenous
MR: Magnetic resonance
mPFS: Median progression-free survival
mOS: Median overall survival
NCI-CTC: National Cancer Institute Common Toxicity Criteria
NENs: Neuroendocrine neoplasms
NGEP: Non-gastroenteropancreatic
NR: Not reached
ORR: Overall response rate
PET/CT: Positron emission tomography and computed tomography
pNENs: Pancreatic neuroendocrine neoplasms
PPGL: Pheochromocytoma/paraganglioma
PRRT: Peptide receptor radionuclide therapy
RCT: Randomized clinical trial
RECIST v1.1: Response Evaluation Criteria in Solid Tumors version 1.1
SEMNIM: Spanish Society of Nuclear Medicine and Molecular Imaging
SEEN: Spanish Society of the Endocrinology and Nutrition
SRI: SSTR imaging
SSA: Somatostatin analogs
SSTRs: Somatostatin receptors
Y-90: Yttrium-90

## Declarations

### Disclaimers

The SEPTRALU registry (SEMNIM-SER-2021-01) is promoted by Spanish Society of Nuclear Medicine and Molecular Imaging (SEMNIM) in collaboration with the Spanish Society of Endocrinology and Nutrition (SEEN).
