# Supplementary material for: Efficacy of [177Lu]Lu-DOTATATE in metastatic neuroendocrine neoplasms of different locations: data from the SEPTRALU study
Source: Eur J Nucl Med Mol Imaging. 2023 Mar 6;50(8):2486–500. doi: 10.1007/s00259-023-06166-8 (PMC10250456; doi:10.1007/s00259-023-06166-8)
Supplement: Supplementary file 1 — Supplementary file1 (DOCX 18 KB) [file 259_2023_6166_MOESM1_ESM.docx]

**Supplementary Materials, Annex Table 1.** Baseline characteristics aggregated into GEP-NENs and NGEP-NENs.

| **Baseline Characteristic** | **All patients, N=522 (100%)** | **GEP-NEN,**  **N=390 (100%)** | **NGEP-NEN,**  **N=132 (100%)** |
| --- | --- | --- | --- |
| **Gender**  Men  Women | 314 (60.2)  208 (39.8) | 231 (59.2)  159 (40.8) | 83 (62.9)  49 (37.1) |
| **Age (years)**, median (range) | 60 (21-88) | 61 (21-88) | 59 (21-83) |
| **ECOG performance status**  0  1  2  >2  Unknown | 256 (49.0)  210 (40.2)  37 (7.1)  4 (0.8)  15 (2.9) | 202 (51.8)  148 (37.9)  27 (6.9)  2 (0.5)  11 (2.8) | 54 (40.9)  62 (47.0)  10 (7.6)  2 (1.5)  4 (3.0) |
| **Primary tumor site**  Pancreatic  Small intestine  Colorectum  Esophagogastric  Other GEP  Unknown primary of digestive origin  Lung  Pheochromocytoma  Paraganglioma  Thymus  Thyroid  Unknown origin  Other NGEP | 182 (34.8)  153 (29.3)  24 (4.1)  9 (1.7)  11 (2.1)  11 (2.1)  56 (10.7)  9 (1.7)  22 (4.2)  4 (0.7)  2 (0.3)  32 (6.1)  7 (1.3) | 182 (46.7)  153 (39.2)  24 (6.2)  9 (2.3)  11 (2.8)  11 (2.8)  -  -  -  -  -  -  - | -  -  -  -  -  -  56 (42.4)  9 (6.8)  22 (16.7)  4 (3.0)  2 (1.5)  32 (24.2)  7 (5.3) |
| **Ki-67, median (range)**  Missing | 5 (0-80)  95 (18.2) | 5 (1-80)  56 (14.4) | 7 (0-77)  39 (29.5) |
| **Grade (WHO 2017)**  NET G1  NET G2  NET G3  NEC G3 | 178 (34.1)  292 (55.9)  42 (8.0)  10 (1.9) | 138 (35.4)  216 (55.4)  30 (7.7)  6 (1.5) | 40 (30.3)  76 (57.6)  12 (9.1)  4 (3.0) |
| **Hormonal syndrome** | 169 (32.4) | 125 (32.1) | 44 (33.3) |
| **Localization of metastases**  Liver  Lymph nodes  Peritoneum  Bone  Lung  Other | 436 (83.5)  286 (54.8)  90 (17.2)  145 (27.8)  48 (9.2)  78 (14.9) | 350 (89.7)  208 (53.3)  77 (19.7)  77 (19.7)  20 (5.1)  52 (13.3) | 86 (65.2)  78 (59.1)  13 (9.8)  68 (51.5)  28 (21.2)  26 (19.7) |
| **Prior surgery**  Primary tumor  Metastases | 281 (53.8)  121 (23.2) | 208 (53.3)  104 (26.7) | 73 (55.3)  17 (12.9) |
| **Number of prior systemic treatments**  0  1  2  >2 | 22 (4.2)  184 (35.2)  144 (29.7)  161 (30.8) | 5 (1.3)  150 (38.5)  119 (30.5)  116 (29.7) | 17 (12.9)  34 (25.8)  36 (27.3)  45 (34.1) |
| **Prior systemic treatments**  Somatostatin analogues  Chemotherapy  Everolimus  Sunitinib  Other tyrosin quinase inhibitor | 477 (91.4)  144 (27.6)  224 (42.9)  104 (19.9)  54 (10.3) | 371 (95.1)  96 (24.6)  169 (43.3)  83 (21.3)  36 (9.2) | 106 (80.3)  48 (36.4)  55 (41.7)  21 (15.9)  18 (13.6) |
| **Prior locoregional and ablative therapies** | 77 (14.8) | 69 (17.7) | 8 (6.1) |
| **Median time from initial diagnosis to PRRT, months (range)** | 40.6 (0-288) | 40.3 (0-288) | 41 (1.8-273) |
| **Median time from most recent progression until PRRT, months (range)** | 2.3 (0-92.1) | 2.3 (0-92) | 2.5 (0-2) |
| **SRI, Krenning scale**  2  3  4 | 44 (8.4)  395 (75.7)  83 (15.9) | 28 (7.2)  304 (77.9)  58 (14.9) | 16 (12.1)  91 (68.9)  25 (18.9) |
| **[^18^F]F-FDG PET-CT**  Not done  Consistent with SRI  Not concordant | 383 (73.4)  50 (9.6)  89 (17.0) | 302 (77.4)  24 (6.2)  64 (16.4) | 81 (61.4)  26 (19.7)  25 (18.9) |

Abbreviations: GEP, gastroenteropancreatic; NGEP, no gastroenteropancreatic; ECOG, Eastern Cooperative Oncology Group; WHO, World Health Organization; PRRT, peptide receptor radionuclide therapy; PET,  Positron emission tomography; SRI, somatostatin receptor-based imaging; 18F-fluorodeoxyglucose position emission tomography-computed tomography; NET, neuroendocrine tumor; NEC, neuroendocrine carcinoma.
