# Supplementary material for: Efficacy of [177Lu]Lu-DOTATATE in metastatic neuroendocrine neoplasms of different locations: data from the SEPTRALU study
Source: Eur J Nucl Med Mol Imaging. 2023 Mar 6;50(8):2486–500. doi: 10.1007/s00259-023-06166-8 (PMC10250456; doi:10.1007/s00259-023-06166-8)
Supplement: Supplementary file 2 — Supplementary file2 (DOCX 16 KB) [file 259_2023_6166_MOESM2_ESM.docx]

**Supplementary Materials, Annex Table 2A. Correlation of the Krenning score with the line of treatment.**

| **Prior lines of therapy** | **Krenning 2,**  **N (%)** | **Krenning 3,**  **N (%)** | **Krenning 4,**  **N (%)** | **Total,**  **N (%)** |
| --- | --- | --- | --- | --- |
| **0** | 3 (13.6) | 14 (63.6) | 5 (22.7) | 22 (100.0) |
| **1** | 19 (10.3) | 137 (74.5) | 28 (15.2) | 184 (100) |
| **2** | 9 (5.8) | 117 (75.5) | 29 (18.7) | 155 (100) |
| **>2** | 13 (8.1) | 127 (78.9) | 21 (13.0) | 161 (100) |

χ2=5.7, degrees of freedom= 6, p-value=0.451

**Supplementary Materials, Annex Table 2B. Correlation of the Krenning score with the histologic grade.**

| **Grade** | **Krenning 2,**  **N (%)** | **Krenning 3,**  **N (%)** | **Krenning 4,**  **N (%)** | **Total,**  **N (%)** |
| --- | --- | --- | --- | --- |
| **1** | 15 (8.4) | 141 (79.2) | 22 (12.4) | 178 (100) |
| **2** | 21 (7.2) | 224 (76.7) | 47 (16.1) | 292 (100) |
| **3** | 8 (15.4) | 30 (57.7) | 14 (26.9) | 52 (100) |

χ2=11.4, degrees of freedom=4, **p-value=0.0219**

**Supplementary Materials, Annex Table 2C. Progression-free survival according to the Krenning score in NENs grade 3 (n=52).**

| **Krenning score** | **N/n** | **Median PFS (months)** | **95% CI** |
| --- | --- | --- | --- |
| 2 | 8/8 | 8.86 | 7.56-NR |
| 3 | 30/23 | 12.89 | 7.43-24.2 |
| 4 | 14/6 | 26.93 | 11.77-NR |

NR, not reached; PFS, progression-free survival; CI, confidence interval.

**Supplementary Materials, Annex Table 2D. Association between the Krenning Score and Diagnostic Method.**

|  | **Krenning 2,**  **N (%)** | **Krenning 3,**  **N (%)** | **Krenning 4,**  **N (%)** | **Total,**  **N (%)** |
| --- | --- | --- | --- | --- |
| **68Ga-DOTATOC** | 4 (7.0) | 34 (59.6) | 19 (33.3) | 57 (100.0) |
| **SSTR scintigraphy** | 40 (8.6) | 361 (77.8) | 64 (13.8) | 464 (100) |
| **Total** | 44 (8.4) | 395 (75.6) | 83 (15.9) | 522 (100) |

χ2=14.481, degrees of freedom=2, p-value<0.0001
