# Supplementary material for: Efficacy of [177Lu]Lu-DOTATATE in metastatic neuroendocrine neoplasms of different locations: data from the SEPTRALU study
Source: Eur J Nucl Med Mol Imaging. 2023 Mar 6;50(8):2486–500. doi: 10.1007/s00259-023-06166-8 (PMC10250456; doi:10.1007/s00259-023-06166-8)
Supplement: Supplementary file 3 — Supplementary file3 (DOCX 18 KB) [file 259_2023_6166_MOESM3_ESM.docx]

**Supplementary Materials, Annex Table 3A. Response rate based on the number of previous lines in pNENs.**

| **N. of previous lines** | **PD, N (%)** | **SD, N (%)** | **PR, N (%)** | **CR, N (%)** | **Not evaluated, N (%)** | **Total, N (%)** |
| --- | --- | --- | --- | --- | --- | --- |
| **0-1** | 3 (5.1) | 23 (39.0) | 27 (45.8) | 1 (1.7) | 5 (8.5) | 59 (100) |
| **2** | 9 (16.4) | 15 (27.3) | 22 (40.0) | 0 | 9 (16.4) | 55 (100) |
| **>2** | 12 (17.6) | 29 (42.6) | 17 (25.0) | 0 | 10 (14.7) | 68 (100) |
| **All** | 24 (13.2) | 67 (36.8) | 66 (36.3) | 1 (0.5) | 24 (13.2) | 182 (100) |
| Χ2=14.1, degrees of freedom = 8, p-value= 0.0786 | | | | | | |

**Supplementary Materials, Annex Table 3B.** **Response rate based on the number of previous lines in midgut NENs.**

| **N. of previous lines** | **PD, N (%)** | **SD, N (%)** | **PR, N (%)** | **CR, N (%)** | **Not evaluated, N (%)** | **Total, N (%)** |
| --- | --- | --- | --- | --- | --- | --- |
| **0-1** | 4 (5.4) | 39 (52.7) | 15 (20.3) | 0 | 16 (21.6) | 74 (100) |
| **2** | 3 (6.2) | 26 (54.2) | 13 (27.1) | 1 (2.1) | 5 (10.4) | 48 (100) |
| **>2** | 1 (3.8) | 16 (61.5) | 6 (23.1) | 0 | 3 (11.5) | 26 (100) |
| **All** | 8 (5.4) | 81 (54.7) | 34 (23%) | 1 (0.7) | 24 (16.2) | 148 (100) |
| Χ2=5.81, degrees of freedom = 8, p-value= 0.668 | | | | | | |

**Supplementary Materials, Annex Table 3C**. **Response rate based on the number of previous lines in BP-NENs.**

| **N. of previous lines** | **PD, N (%)** | **SD, N (%)** | **PR, N (%)** | **CR, N (%)** | **Not evaluated, N (%)** | **Total, N (%)** |
| --- | --- | --- | --- | --- | --- | --- |
| **0-1** | 0 | 7 (50.0) | 6 (42.9) | 0 | 1 (7.1) | 14 (100) |
| **2** | 4 (19.0) | 10 (47.6) | 4 (19.0) | 0 | 3 (14.3) | 21 (100) |
| **>2** | 7 (33.3) | 7 (33.3) | 4 (19.0) | 0 | 3 (14.3) | 21 (100) |
| **All** | 11 (19.6) | 24 (42.9) | 14 (25.0) | 0 | 7 (12.5) | 56 (100) |
| Χ2=7.5, degrees of freedom = 4, p-value= 0.107 | | | | | | |

**Supplementary Materials, Annex Table 3D.** **Response rate based on the number of previous lines in PPGLs.**

| **N. of previous lines** | **PD, N (%)** | **SD, N (%)** | **PR, N (%)** | **CR, N (%)** | **Not evaluated, N (%)** | **Total, N (%)** |
| --- | --- | --- | --- | --- | --- | --- |
| **0-1** | 1 (5.6) | 11 (61.1) | 4 (22.2) | 0 | 2 (11.1) | 18 (100.0) |
| **2** | 1 (20.0) | 4 (80.0) | 0 | 0 | 0 | 5 (100.0) |
| **>2** | 2 (25.0) | 2 (25.0) | 1 (12.5) | 0 | 3 (37.5) | 8 (100.0) |
| **All** | 4 (12.9) | 17 (54.8) | 5 (16.1) | 0 | 5 (16.1) | 31 (100.0) |
| Χ2=8.4, degrees of freedom = 6, p-value= 0.203 | | | | | | |

**Supplementary Materials, Annex Table 3E.** **Response rate based on the number of previous lines in other GEP-NENs.**

| **N. of previous lines** | **PD, N (%)** | **SD, N (%)** | **PR, N (%)** | **CR, N (%)** | **Not evaluated, N (%)** | **Total, N (%)** |
| --- | --- | --- | --- | --- | --- | --- |
| **0-1** | 0 | 8 (36.4) | 4 (18.2) | 0 | 10 (45.5) | 22 (100.0) |
| **2** | 3 (18.8) | 8 (50.0) | 5 (31.2) | 0 | 0 | 16 (100.0) |
| **>2** | 4 (18.2) | 8 (36.4) | 8 (36.4) | 0 | 2 (9.1) | 22 (100.0) |
| **All** | 7 (11.7) | 24 (40.0) | 17 (28.3) | 0 | 12 (20.0) | 60 (100.0) |
| Χ2=3.6, degrees of freedom = 4, p-value= 0.459 | | | | | | |

**Supplementary Materials, Annex Table 3F.** **Response rate based on the number of previous lines in other NGEP-NENs.**

| **N. of previous lines** | **PD, N (%)** | **SD, N (%)** | **PR, N (%)** | **CR, N (%)** | **Not evaluated, N (%)** | **Total, N (%)** |
| --- | --- | --- | --- | --- | --- | --- |
| **0-1** | 3 (15.8) | 9 (47.4) | 2 (10.5) | 1 (5.3) | 4 (21.1) | 19 (100) |
| **2** | 0 | 5 (50.0) | 4 (40.0) | 0 | 1 (10.0) | 10 (100) |
| **>2** | 5 (31.2) | 4 (25.0) | 5 (31.2) | 0 | 2 (12.5) | 16 (100) |
| **All** | 8 (17.8) | 18 (40.0) | 11 (24.4) | 1 (2.2) | 7 (15.6) | 45 (100) |
| Χ2=9.6, degrees of freedom = 8, p-value= 0.287 | | | | | | |

Abbreviations: pNEN, pancreatic neuroendocrine neoplasm; BP-NEN, bronchopulmonary neuroendocrine neoplasm; PPGL, pheochromocytoma and paraganglioma; NEN, neuroendocrine neoplasia; GEP, gastroenteropancreatic; NGEP, no gastroenteropancreatic; PD, progression disease; SD, stable disease; PR, partial response; CR, complete response, N., number.
