# Supplementary material for: Efficacy of [177Lu]Lu-DOTATATE in metastatic neuroendocrine neoplasms of different locations: data from the SEPTRALU study
Source: Eur J Nucl Med Mol Imaging. 2023 Mar 6;50(8):2486–500. doi: 10.1007/s00259-023-06166-8 (PMC10250456; doi:10.1007/s00259-023-06166-8)
Supplement: Supplementary file 4 — Supplementary file4 (DOCX 13 KB) [file 259_2023_6166_MOESM4_ESM.docx]

**Annex Table 4.** **Response assessment according to tumor grade in subjects with measurable and response-assessable disease.**

| **Grade (WHO 2017)** | **PD, N (%)** | **SD, N (%)** | **PR, N (%)** | **CR, N (%)** | **Total, N (%)** |
| --- | --- | --- | --- | --- | --- |
| **NET G1** | 18 (12.0) | 76 (50.7) | 54 (36.0) | 2 (1.3) | 150 (100) |
| **NET G2** | 32 (13.0) | 138 (56.1) | 75 (30.5) | 1 (0.4) | 246 (100) |
| **NET G3** | 3 (30.0) | 3 (30.0) | 4 (40.0) | 0 | 10 (100) |
| **NEG G3** | 9 (24.3) | 14 (37.8) | 14 (37.8) | 0 | 37 (100) |
| **Total** | 62 (14.0) | 231 (52.1) | 147 (33.2) | 3 (0.7) | 443 (100) |

Abbreviations: NET, neuroendocrine tumor, NEC, neuroendocrine carcinoma; WHO, World Health Organization; PD, progression disease; SD, stable disease; PR, partial response; CR, complete response.

Note: Response was evaluated using RECIST v1.1 criteria.
