# Supplementary material for: Efficacy of [177Lu]Lu-DOTATATE in metastatic neuroendocrine neoplasms of different locations: data from the SEPTRALU study
Source: Eur J Nucl Med Mol Imaging. 2023 Mar 6;50(8):2486–500. doi: 10.1007/s00259-023-06166-8 (PMC10250456; doi:10.1007/s00259-023-06166-8)
Supplement: Supplementary file 5 — Supplementary file5 (DOCX 15 KB) [file 259_2023_6166_MOESM5_ESM.docx]

**Supplementary Materials, Annex Table 5A. Progression-free survival based on the number of previous lines in pNENs.**

| **N. of previous lines** | **N/n** | **Median PFS (months)** | **95% CI** |
| --- | --- | --- | --- |
| **0-1** | 59/16 | 66.2 | 25.0-NR |
| **2** | 55/32 | 17.0 | 14.7-27.2 |
| **>2** | 68/42 | 14.6 | 11.9-28.6 |

**Supplementary Materials, Annex Table 5B. Progression-free survival based on the number of previous lines in midgut NENs.**

| **N. of previous lines** | **N/n** | **Median PFS (months)** | **95% CI** |
| --- | --- | --- | --- |
| **0-1** | 74/19 | 50.8 | 27.1-NR |
| **2** | 48/19 | 29.3 | 22.7-NR |
| **>2** | 26/15 | 23.3 | 15.2-NR |

**Supplementary Materials, Annex Table 5C. Progression-free survival based on the number of previous lines in BP-NENs.**

| **N. of previous lines** | **N/n** | **Median PFS (months)** | **95% CI** |
| --- | --- | --- | --- |
| **0-1** | 14/7 | 33.1 | 18.18-NR |
| **2** | 21/11 | 16.8 | 8.38-NR |
| **>2** | 21/16 | 17.3 | 9.86-NR |

**Supplementary Materials, Annex Table 5D. Progression-free survival based on the number of previous lines in PPGLs.**

| **N. of previous lines** | **N/n** | **Median PFS (months)** | **95% CI** |
| --- | --- | --- | --- |
| **0-1** | 18/5 | 45.27 | 30.58-NR |
| **2** | 5/3 | 12.43 | 9.73-NR |
| **>2** | 8/7 | 9.34 | 7.53-NR |

**Supplementary Materials, Annex Table 5E. Progression-free survival based on the number of previous lines in other GEP-NENs.**

| N. of previous lines | N/n | Median PFS (months) | 95% CI |
| --- | --- | --- | --- |
| 0-1 | 22/6 | NA | 42.31-NR |
| 2 | 16/7 | 20.0 | 12.85-NR |
| >2 | 22/16 | 16.6 | 9.93-NR |

**Supplementary Materials, Annex Table 5F. Progression-free survival based on the number of previous lines in other NGEP-NENs.**

| N. of previous lines | N/n | Median PFS (months) | 95% CI |
| --- | --- | --- | --- |
| 0-1 | 19/7 | 19.1 | 19.07-NR |
| 2 | 10/4 | 36.6 | 10.75-NR |
| >2 | 16/13 | 16.2 | 9.86-NR |

Abbreviations: N/n, sample/events; PFS, progression-free survival; CI, confidence interval; pNEN, pancreatic neuroendocrine neoplasm; BP-NEN, bronchopulmonary neuroendocrine neoplasm; PPGL, pheochromocytoma and paraganglioma; NEN, neuroendocrine neoplasm: GEP, gastroenteropancreatic; NGEP, no gastroenteropancreatic; PD, progression disease: SD, stable disease; PR, partial response; CR, complete response, N., number.
