# Supplementary material for: Efficacy of [177Lu]Lu-DOTATATE in metastatic neuroendocrine neoplasms of different locations: data from the SEPTRALU study
Source: Eur J Nucl Med Mol Imaging. 2023 Mar 6;50(8):2486–500. doi: 10.1007/s00259-023-06166-8 (PMC10250456; doi:10.1007/s00259-023-06166-8)
Supplement: Supplementary file 6 — Supplementary file6 (DOCX 17 KB) [file 259_2023_6166_MOESM6_ESM.docx]

**Supplementary Materials, Annex Table 6A.** Progression-free survival based on tumor site in grade 1 and 2 NENs.

| **Tumor site** | **N/n** | **Median PFS (months)** | **95% CI** |
| --- | --- | --- | --- |
| **pNENs** | 154/70 | 23.4 | 18.2-45.2 |
| **Midgut NENs** | 143/49 | 31.3 | 25.7-NR |
| **BP-NENs** | 50/31 | 17.6 | 13.6-NR |
| **PPGLs** | 29/14 | 30.6 | 14.4-NR |
| **Other GEP-NENs** | 57/26 | 32.8 | 18.7-NR |
| **Other NGEP-NENs** | 37/18 | 21.0 | 11.1-NR |

Abbreviations: N/n, sample/events; PFS, progression-free survival; CI, confidence interval; NR, not reached; pNEN, pancreatic neuroendocrine neoplasm; BP-NEN, bronchopulmonary neuroendocrine neoplasm; PPGL, pheochromocytoma and paraganglioma; NEN, neuroendocrine neoplasia: GEP, gastroenteropancreatic; NGEP, no gastroenteropancreatic.

**Supplementary Materials, Annex Table 6B.** Progression-free survival based on grade (WHO 2017).

| **Grade** | **N/n** | **Median PFS (months)** | **95% CI** |
| --- | --- | --- | --- |
| **NET G1** | 178/72 | 30.6 | 25.41-42.3 |
| **NET G2** | 292/136 | 21.3 | 19.30-31.6 |
| **NET G3** | 42/29 | 12.9 | 8.71-26.9 |
| **NEC G3** | 10/8 | 17.1 | 9.90-NR |

Abbreviations: WHO, World Health Organization; N/n, sample/events; PFS, progression-free survival; CI, confidence interval; NET, neuroendocrine tumor; NEC, neuroendocrine carcinoma; NR, not reached

**Supplementary Materials, Annex Table 6C.** Progression-free survival determined by assessment method.

|  | **N/n** | **Median PFS (months)** | **95% CI** |
| --- | --- | --- | --- |
| **[^68^Ga]Ga-DOTATOC, any Krenning** | 57/22 | 19.1 | 14.6-NR |
| **SSTR scintigraphy, any Krenning** | 465/222 | 25.4 | 21.0-29.4 |
| Logrank test, χ2= 0.5 on 1 degrees of freedom, p= 0.5 | | | |

Abbreviations: N/n, sample/events; PFS, progression-free survival; CI, confidence interval; NR, not reached; SSTRs: somatostatin receptors.

**Supplementary Materials, Annex Table 6D.** Progression-free survival determined by uptake intensity and assessment method.

|  | **N/n** | **Median PFS (months)** | **95% CI** |
| --- | --- | --- | --- |
| **Any method + Kreening score 2** | 44/24 | 19.8 | 9.86-NR |
| **[^68^Ga]Ga-DOTATOC + Krenning score 3** | 34/13 | 19.7 | 14.7-NR |
| **[^68^Ga]Ga-DOTATOC + Krenning score 4** | 19/6 | 19.1 | 9.8-NR |
| **SSTR scintigraphy + Krenning score 3** | 361/169 | 24.3 | 20.5-31.5 |
| **SSTR scintigraphy + Krenning score 4** | 64/32 | 28.3 | 21.3-49.2 |
| Logrank test, χ2= 2 on 4 degrees of freedom, p= 0.7 | | | |

Abbreviations: N/n, sample/events; PFS, progression-free survival; CI, confidence interval; NR, not reached; SSTRs; somatostatin receptors.
