# Supplementary material for: Efficacy of [177Lu]Lu-DOTATATE in metastatic neuroendocrine neoplasms of different locations: data from the SEPTRALU study
Source: Eur J Nucl Med Mol Imaging. 2023 Mar 6;50(8):2486–500. doi: 10.1007/s00259-023-06166-8 (PMC10250456; doi:10.1007/s00259-023-06166-8)
Supplement: Supplementary file 7 — Supplementary file7 (DOCX 14 KB) [file 259_2023_6166_MOESM7_ESM.docx]

**Supplementary Materials, Annex Table 7. Hematological toxicity by lines of prior treatment.**

| **N. of previous lines** | **Grade 0, N (%)** | **Grade 1, N (%)** | **Grade 2, N (%)** | **Grade 3, N (%)** | **Grade 4, N (%)** | **Total,**  **N (%)** |
| --- | --- | --- | --- | --- | --- | --- |
| **0** | 15 (5.1) | 109 (37.1) | 84 (28.6) | 86 (29.3) | 0 | 294 (100) |
| **1** | 5 (5.2) | 31 (32.3) | 31 (32.3) | 29 (30.2) | 0 | 96 (100) |
| **2** | 0 | 10 (28.6) | 11 (31.4) | 14 (40.0) | 0 | 35 (100) |
| **3** | 0 | 5 (25.0) | 7 (35.0) | 8 (40.0) | 0 | 20 (100) |
| **>3** | 0 | 1 (20.0) | 3 (60.0) | 1 (20.0) | 0 | 5 (100) |
| **NA** | 2 (2.8) | 28 (38.9) | 19 (26.4) | 23 (31.9) | 0 | 72 (100) |
| **All** | 22 (4.2) | 184 (35.2) | 155 (29.7) | 161 (30.8) | 0 | 522 (100) |
| Χ2=8.9, degrees of freedom = 12, p-value= 0.708 | | | | | | |

Toxicity based on NCI-CTC criteria; NA, not available
