# Supplementary material for: Efficacy of [177Lu]Lu-DOTATATE in metastatic neuroendocrine neoplasms of different locations: data from the SEPTRALU study
Source: Eur J Nucl Med Mol Imaging. 2023 Mar 6;50(8):2486–500. doi: 10.1007/s00259-023-06166-8 (PMC10250456; doi:10.1007/s00259-023-06166-8)
Supplement: Supplementary file 8 — Supplementary file8 (PDF 25 KB) [file 259_2023_6166_MOESM8_ESM.pdf]

Annex Figure 1. Landmark analysis survival curves for overall survival from the 12 month-point

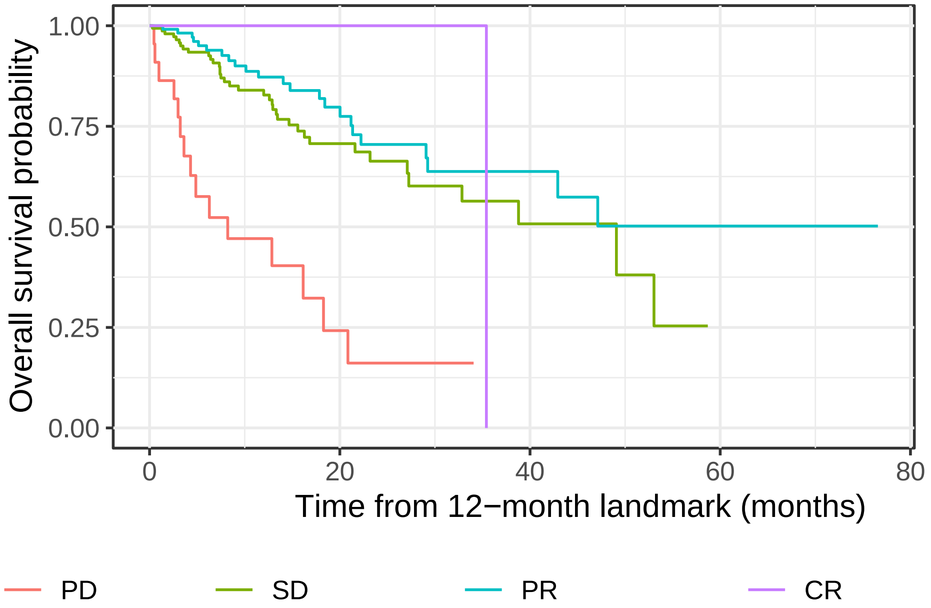

At Risk

	0	20	40	60	80
PD	22	3	0	0	0
SD	158	37	7	0	0
PR	122	35	12	2	0
CR	3	2	0	0	0

Events

	0	20	40	60	80
PD	0	14	15	15	15
SD	0	29	35	37	37
PR	0	15	21	23	23
CR	0	0	1	1	1

Concordance= 0.646 (standard error = 0.034). Abbreviations: PD= progressive disease, SD= stable disease, PR= partial response, CR= complete response.
